# Choroidal infarction in a glaucoma patient with Flammer syndrome: a case report with a long term follow-up

**DOI:** 10.1186/s12886-017-0416-4

**Published:** 2017-03-14

**Authors:** Barbara Terelak-Borys, Iwona Grabska-Liberek, Anita Piekarniak-Wozniak, Katarzyna Konieczka

**Affiliations:** 1 Department of Ophthalmology, Centre of Postgraduate Medical Education, Czerniakowska str. 231, 01-416 Warsaw, Poland; 2 Department of Ophthalmology, University of Basel, Mittlere str. 91, CH 4012 Basel, Switzerland

**Keywords:** Choroidal infarction, Flammer syndrome, Primary vascular dysregulation, Glaucoma

## Abstract

**Background:**

We present a long term follow-up of a young female patient with choroidal infarction, primary open angle glaucoma and Flammer syndrome. The patient had no classical risk factors for vascular occlusions, except for the presence of Flammer syndrome. The essential feature of this syndrome is primary vascular dysregulation, sometimes including vasospasm. The vessels of affected people respond more intensely to a number of stimuli, such as coldness or emotional stress. Any organ can be involved, including parts of the eye. The dense autonomic innervation of the choroidal vessels predisposes them particularly to vasospasms.

**Case presentation:**

The patient was originally referred to our centre because of a deep unilateral paracentral scotoma with the presumptive diagnosis of a normal tension glaucoma. The discrepancy between the visual field defect and the optic nerve head morphology, however, led us to a vascular evaluation by a simultaneous fluorescein/indocyanine green angiography. This revealed an antecedent choroidal infarction that explained the visual field scotoma and the retinal nerve fibre layer defect in the corresponding area. During the follow-up period of 11 years, the patient also developed bilateral glaucomatous optic neuropathy despite a well-controlled intraocular pressure.

**Conclusions:**

We hypothesise that in the patient presented here, the Flammer syndrome contributed to both the acute unilateral choroidal infarction and to the chronic development of bilateral glaucomatous optic neuropathy.

## Background

Occlusions of ocular vessels are serious and sight threatening events. They occur particularly in elderly people with cardiovascular risk factors. However, such events can also occur, although much less frequently, in younger people without classical cardiovascular risk factors and in the absence of other diseases. Functional reversible vasoconstrictions (vasospasms) are considered possible mechanisms for such infarctions in younger people.

If vasospasms are present in several organs of the same subject, simultaneously or sequentially, the term vasospastic syndrome is used. The more general term vascular dysregulation encompasses not only spasms but any form of inappropriate constriction and dilatation. If vascular dysregulation is not caused by a disease, it is called primary vascular dysregulation (PVD). Subjects with PVD not only have vascular dysregulation but also other signs and symptoms, as summarized in a recent review [1, 2]. Therefore, the combination of PVD with this cluster of associated vascular and nonvascular signs and symptoms is called Flammer syndrome (FS) [2]. The main vascular feature of FS is a predisposition to respond differently to a number of stimuli such as coldness or emotional stress [3, 4].

PVD can affect any organ, including the eye [1, 2], leading to a variety of effects depending on the intensity and duration of the resulting hypoxia. In subjects with PVD, vascular occlusions (including Susac syndrome, anterior ischemic optic neuropathy, and myocardial infarctions) can occur, though rarely, at a young age and in the absence of risk factors for arteriosclerosis. This is particularly true for retinal vein occlusions [1, 2]. Most often PVD is harmless. Unstable blood flow, however, increases local oxidative stress, contributing to the pathogenesis of glaucomatous damage [5–9]. Disturbed autoregulation and fluctuation of ocular perfusion pressure leads to an unstable oxygen supply and therefore to local mitochondrial oxidative stress.

Here we present a young female patient with classical FS who suffered from both an unilateral parapapillary choroidal infarction and a primary open angle glaucoma (POAG).

## Case presentation

**Fig. 1:**
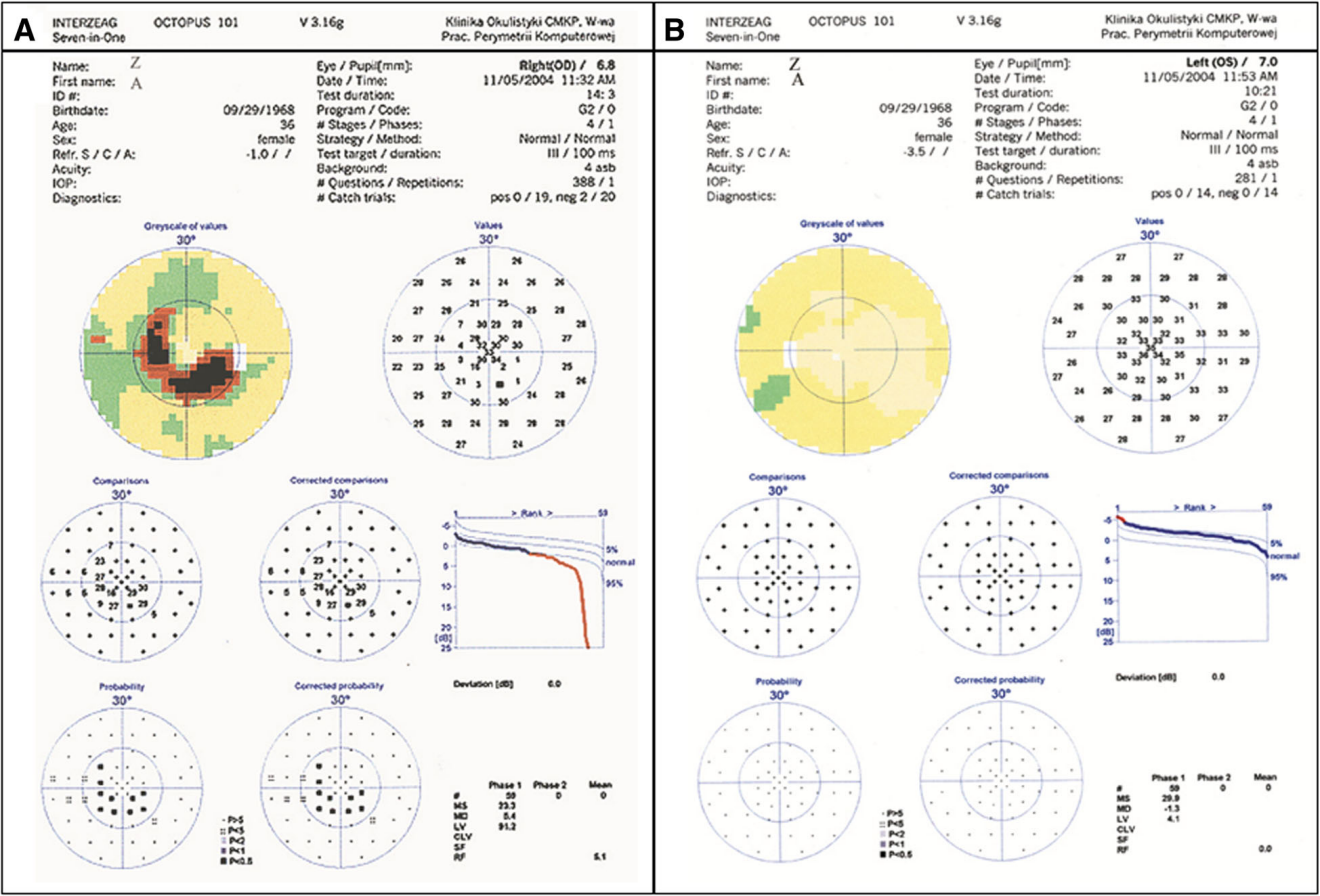
Standard Automated Perimetry (SAP, Octopus 101, G2). Paracentral inferior/nasal scotoma in the right eye (**a**: *right* eye, **b**: *left* eye)

**Fig. 2:**
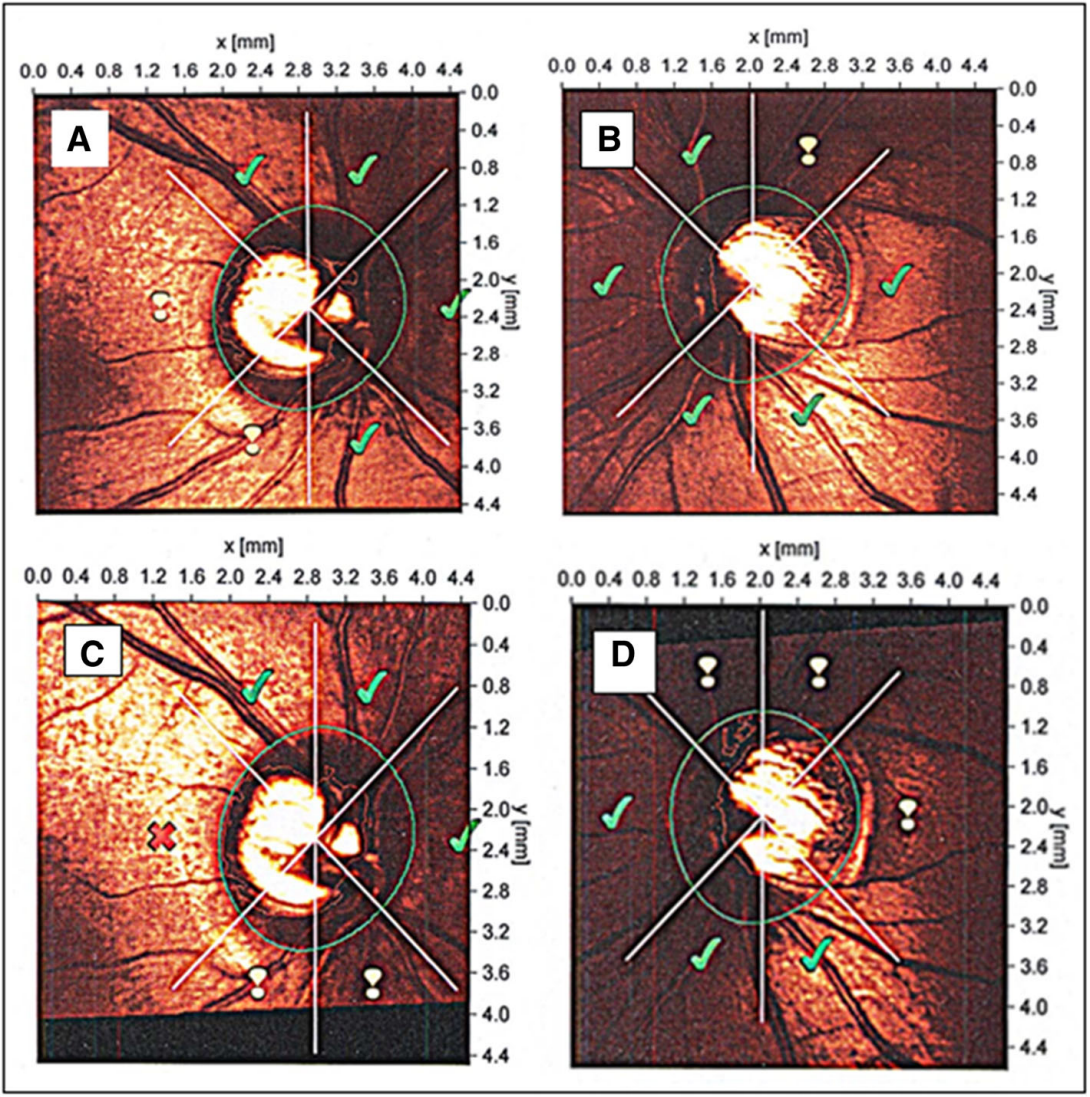
Scanning Laser Tomography (HRT II). Results of initial HRT II examination classified as “Borderline” in both eyes by Moorfields Analysis (**a**: the *right* eye, **b**: the *left* eye). Signs of glaucomatous progression in Moorfields Analysis accompanied by ONH rim loss in both eyes after 11 years of follow-up (**c**: the *right* eye, **d**: the *left* eye)

**Fig. 3:**
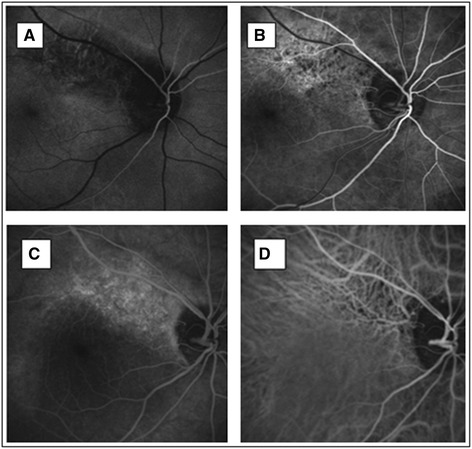
Simultaneous scanning laser fluoresceine/indocyanine green angiography – FA/ICGA (HRA). Choroidal infarction (diminished network of choriocapillaries and retinal pigment epithelium atrophy) in the superior/temporal peripapillary area, corresponding with the visual field defect in the *right* eye. Fundus FA - early phases (**a**, **b**). Simultaneous fundus FA/ICGA – late phase (**c**: FA, **d**: ICGA). More detailed description can be found in the text

A 36-year-old Caucasian woman was referred to our Ophthalmology Department with the suspicion of normal tension glaucoma (NTG) based on a visual field (VF) defect and normal values of intraocular pressure (IOP). Standard automated perimetry (Octopus 101, G2) confirmed, in the right eye (RE), a deep, paracentral scotoma in the inferior/nasal region connected to the blind spot (Mean Damage: MD = 5.4, Loss Variance: LV = 91.2) [10]. The left eye (LE) was unaffected (MD = −1.3, LV = 4.1) (Fig. 1). Up to this date she had not experienced any visual symptoms. Her medical history was also unremarkable, she denied suffering from any diseases or taking any medication. She was slim with classical symptoms of FS like cold hands and feet, low blood pressure and reduced feeling of thirst. She reported having frequent headaches, but not migraine. Nailfold capillaroscopy confirmed vasospasm (spontaneous cessations of blood flow). Systolic blood pressure (BP) fluctuated between 95 to 116 mm Hg, and diastolic BP between 60 to 84 mm Hg. The lowest values occurred at midnight and early in the morning. Family history for glaucoma was negative. Visual acuity (distance in Snellen charts) was 1.0 cc −1.0 D in the RE and 1.0 cc −3.5 D in the LE. Slit lamp examination did not reveal any abnormalities in the anterior segment of both eyes. The anterior chamber angle was open without any abnormalities in either eye. IOP measured by Goldmann applanation tonometry was within normal limits in both eyes: 18–20 mm Hg in the RE and 15–19 mm Hg in the LE. However, the central corneal thickness (CCT) was decreased: 491 μm in the RE and 490 μm in the LE, which indicated that IOP was underestimated. Therefore we corrected IOP by plus 4 mmHg. The cup-to-disc (C/D) ratio of the optic nerve head (ONH) was 0.7 in both eyes. There were no other pathologies in both eyes except enlarged ONH excavations. Scanning laser tomography (HRT II) confirmed symmetric optic nerve disc excavations in both eyes (C/D ratio: 0.674 in the RE and 0.672 in the LE) (Fig. 2a,b). The discs were classified as “borderline” by Moorfield’s analysis, with the suspicion of glaucomatous damage in the temporal/inferior and temporal sectors of the RE and in the temporal/superior sector of the LE. Both discs were relatively large (disc area: RE - 3.390 mm^2^, LE - 3.270 mm^2^). The discrepancy between the VF defect and the ONH appearance and the presence of FS motivated us to evaluate the circulation of ONH and the parapapillary area with a simultaneous fluorescein/indocyanine green angiography (FA/ICGA) with a scanning laser (Heidelberg Retina Angiography) (Fig. 3). Fundus FA demonstrated in the early phase a hypo-fluorescent area in the superior/temporal peripapillary region. Large choroidal vessels were visible here, indicating non-perfusion of the corresponding choriocapillaries (Fig. 3a). In later phases, this area was hyper-fluorescent with “window defects” indicating retinal pigment epithelium (RPE) damage (Fig. 3b,c). The simultaneously performed ICGA revealed diminished perfusion of the choriocapillaries in the area of RPE damage, probably due to atrophy of the corresponding capillaries (Fig. 3d). This area of combined RPE/choriocapillaries atrophy corresponded well with the visual field defect. The angiogram of the LE demonstrate no abnormalities (not shown). The outcome of perimetry combined with the outcome of fundus angiography suggested an antecedent infarction in the parapapillary choroid.

The patient and family history was negative for thrombotic disease or hypercoagulability. Classical risk factors for vascular occlusion were excluded: homocysteine and lipid serum levels were within normal limits, antinuclear antibodies levels in the blood were low and unspecific, and antibodies characteristic of antiphospholipid syndrome (anticardiolipin antibodies and antibodies against β-2-glicoprotein) in the blood were also negative.

As the VF defect was threatening fixation, we introduced an IOP-lowering treatment. First, a therapy with latanoprost was initiated, and later a fixed combination of latanoprost and timolol, but IOP did not diminish satisfactorily. On the latter therapy, IOP fluctuated between 15 to 19 mm Hg in the RE and between 12 to 15 mm Hg in the LE on the diurnal curve, with the highest values in the morning. Thus, the therapy was changed to a fixed combination of bimatoprost and timolol. Under this therapy, IOP measurements did not exceed 11–12 mm Hg in both eyes.

**Fig. 4:**
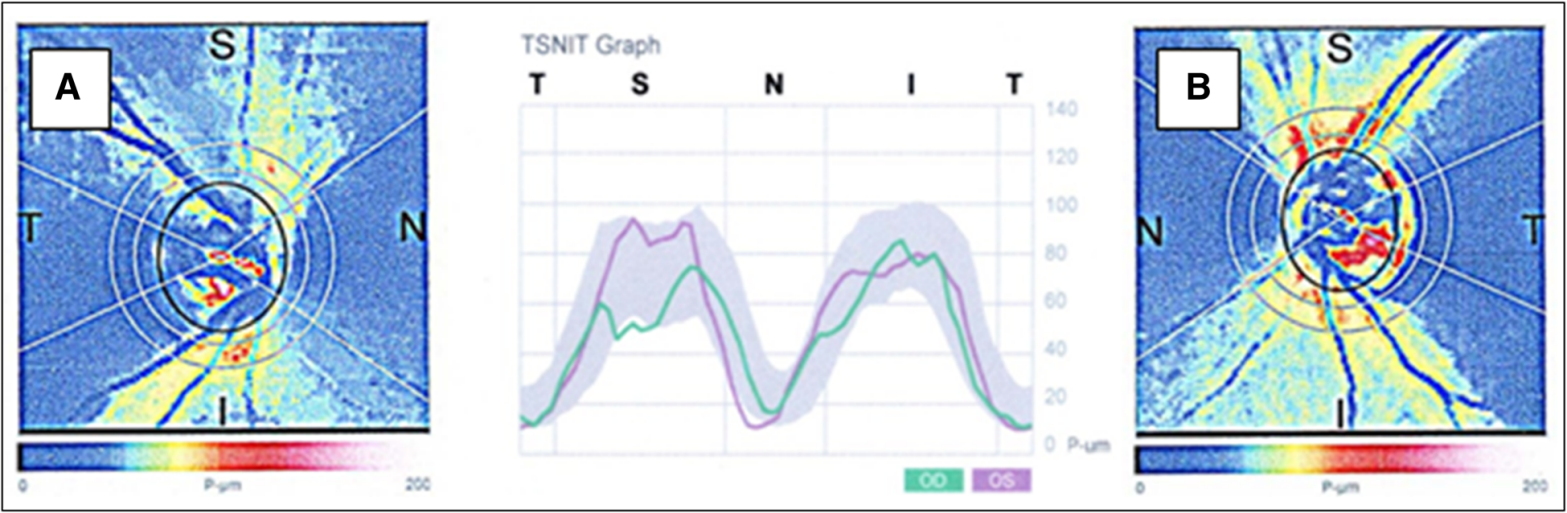
Scanning Laser Polarimetry (GDx ECC). Diminished retinal nerve fibre layer (RNFL) thickness in the superior/temporal peripapillary area, typical for glaucomatous damage in the *right* eye (**a**), corresponding with the area of choroidal infarction and the visual field defect. No detectable RNFL damage is present in the *left* eye (**b**)

The patient received follow-ups over 11 years. The patient developed now classical signs of glaucomatous optic neuropathy. There were signs of neuroretinal rim loss in both eyes. The rim area measured with the HRT II decreased in both eyes (by 0.183 mm^2^ in the RE and by 0.157 mm^2^ in the LE) and more disc sectors were classified as “abnormal” or “borderline” by Moorfield’s analysis (Fig. 2c,d). In addition, scanning laser polarimetry (GDx ECC) revealed diminished retinal nerve fibre layer (RNFL) thickness in the superior/temporal peripapillary area of the RE (Fig. 4a). The visual field index MD fluctuated markedly, which is characteristic for FS subjects. The focal damage (LV), however, remained stable.

## Discussion and Conclusions

We described here a young female patient with classical FS referred to our department with a suspicion of glaucomatous optic neuropathy. However, the deep local VF defect in the RE (Fig. 1) did not correspond to the morphology of the ONH. Both optic discs were only slightly and symmetrically excavated (Fig. 2). There was no notching, which could explain the deep, localized VF scotoma. The angiography revealed combined RPE/choriocapillaries atrophy (Fig. 3) corresponding to the VF defect (Fig. 1), indicating an antecedent choroidal infarction. The examination with GDx revealed reduced RNFL thickness in the corresponding region (Fig. 4).

The patient had no risk factors for arterial occlusions, except a classical FS, which likely predisposed the patient to choroidal infarction. Choroidal infarctions in the context of vasospasms have already been described in the literature. FS has been described to be a risk factor for both retinal arterial and vein occlusions [1], and choroidal infarction [11]. The autoregulatory capacity of the choroid is less efficient than in the retina or ONH [5, 12]. The choroidal vessels are intensively innervated by the autonomic nervous system and are therefore prone to vasospasm. Increased choroidal vasoconstrictive response to sympathetic stimulation was reported in FS subjects [13]. In addition, FS subjects have lower autoregulatory capacity of choroidal circulation than healthy controls [1, 12]. Diminished or even absent autoregulation in the peripapillary choroid has also been described in POAG patients [14]. During the 11-year follow-up period, the patient also developed glaucomatous damage as demonstrated by HRT and GDx examinations (Figs. 2 and 4), in spite of a well-controlled IOP.

FS is considered to be a risk factor for both occlusions of ocular vessels and glaucomatous optic neuropathy (particularly in normal tension glaucoma). The patient presented here had FS, a unilateral nonrecurring choroidal infarction, and a chronic progressive bilateral glaucomatous optic neuropathy. We hypothesise that vasospasms induced the choroidal infarction, and chronic unstable blood flow in the optic nerve head (due to disturbed autoregulation and low blood pressure) increased local oxidative stress and thereby contributed to the development of the glaucomatous optic neuropathy.

The pathogenesis of FS is still unclear. Vascular endothelial dysfunction as well as autonomic nervous system dysregulation could be involved [6–8]. The relationship between the FS and the FS related diseases needs to be established further, in order to promote early diagnosis and targeted prevention in groups at risk.

In summary, we present here a case of a young female patient with FS who experienced an unilateral choroidal infarction despite the absence of classical vascular risk factors, and a bilateral glaucomatous optic neuropathy despite a well-controlled IOP. FS is considered to be a risk factor for both occlusions of ocular vessels and glaucomatous optic neuropathy. The infarction could be induced by vasospasm. We hypothesize that FS also contributed to the development of glaucomatous optic neuropathy by disturbed autoregulation and instability of optic nerve blood flow.

## Abbreviations

BP: Blood pressure
CCT: Central corneal thickness
FA: Fluorescein angiography
FS: Flammer syndrome
ICGA: Indocyanine green angiography
IOP: Intraocular pressure
LE: Left eye
LV: Loss variance
MD: Mean damage
NTG: Normal tension glaucoma
ONH: Optic nerve head
POAG: Primary open angle glaucoma
PVD: Primary vascular dysregulation
RE: Right eye
RNFL: Retinal nerve fibre layer
RPE: Retinal pigment epithelium
VF: Visual field
